# Norovirus on Cruise Ships: A Recurring Sentinel Event

**DOI:** 10.7759/cureus.114832

**Published:** 2026-08-20

**Authors:** Adarsh Pratik Deo, Nisha Thakur, Gursimran Kaur Mohi

**Affiliations:** 1 Virology, Postgraduate Institute of Medical Education and Research (PGIMER), Chandigarh, IND

**Keywords:** cruise ships, gastroenteritis, genomic surveillance, maritime health, molecular epidemiology, norovirus, outbreak investigation, wastewater surveillance

## Abstract

Norovirus remains the leading cause of acute gastroenteritis outbreaks worldwide and is particularly well adapted for transmission aboard cruise ships due to its low infectious dose, prolonged environmental persistence, and rapid person-to-person spread. We reviewed recent outbreaks to highlight recurring norovirus outbreaks and gaps in maritime surveillance. These demonstrate the vulnerability of cruise ships to rapid viral amplification and international dissemination. Current surveillance relies predominantly on symptomatic case reporting, limiting the ability to distinguish between single-source introduction, environmental persistence, foodborne transmission, and emergence of novel recombinant strains. Recent modelling studies have shown that early diagnosis and isolation of symptomatic individuals substantially reduce secondary transmission, yet routine genomic surveillance is rarely implemented onboard. Wastewater-based surveillance offers a practical, non-invasive early warning approach that can be integrated into existing ship infrastructure and complemented by genomic sequencing to identify circulating variants and transmission pathways. Incorporating molecular and wastewater surveillance into routine maritime health programmes would strengthen outbreak detection, improve infection control, and enhance global preparedness for recurrent norovirus outbreaks associated with international cruise travel.

## Editorial

In 2026, three significant norovirus outbreaks were reported aboard the *Star Princess*, *Caribbean Princess*, and *Ambition* between March and May 2026 [1-3]. However, despite recurrent outbreaks, cruise ships continue to be potent amplification environments for norovirus transmission. However, genomic surveillance and outbreak tracking remain restricted [4,5]. This indicates a persistent gap in global maritime infectious disease surveillance, highlighting this continuing public health challenge.

**Figure 1:**
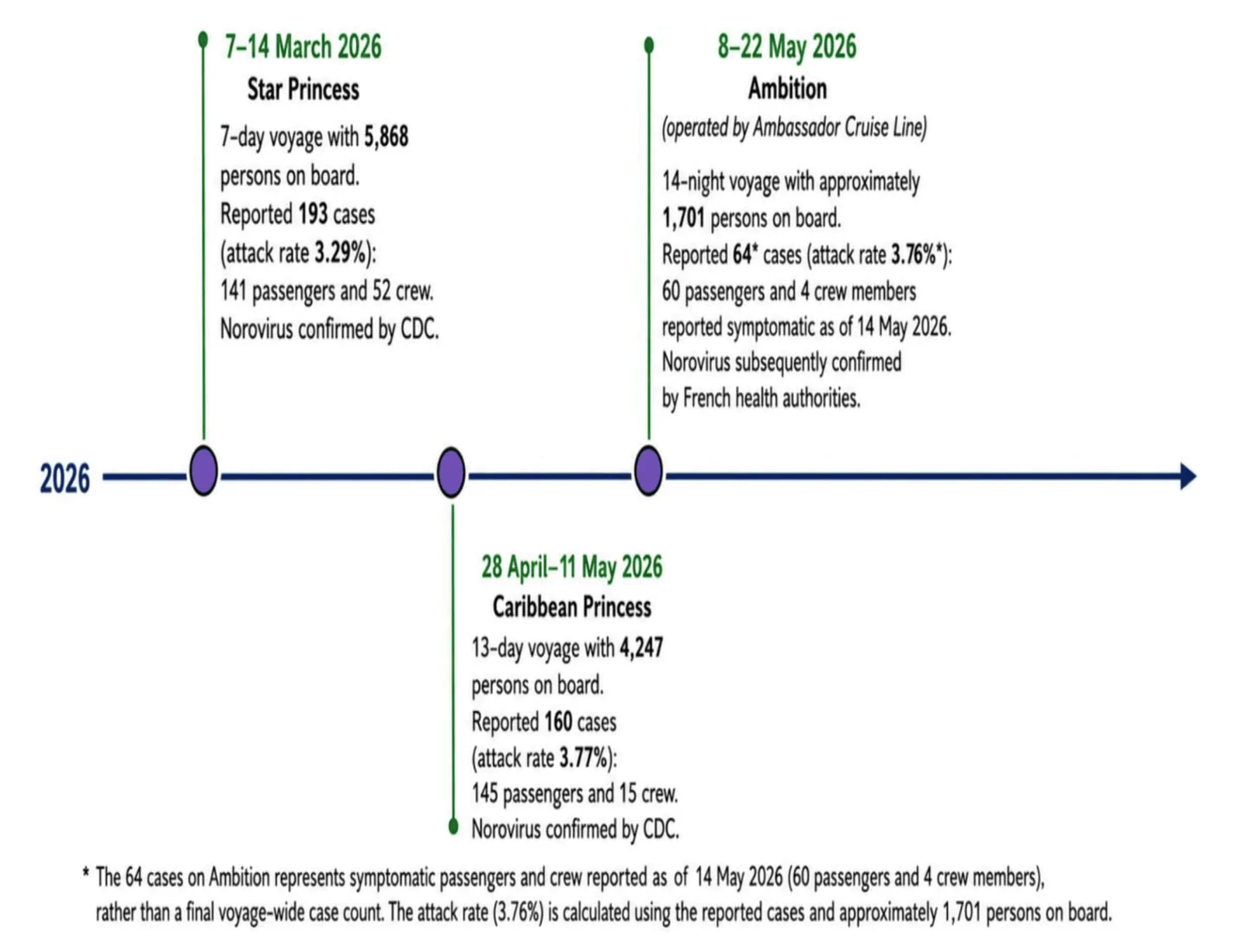
Timeline of norovirus outbreaks on cruise ships (March to May, 2026)

The outbreaks on the three cruise ships highlight how quickly enteric viruses like norovirus can disseminate across international travel networks once introduced into confined maritime settings (Table 1, Figure 1). During a seven-day voyage (March 7-14, 2026), the *Star Princess* recorded 193 cases of gastrointestinal illness among 5,868 passengers and crew, with a 3.29% attack rate. The United States Vessel Sanitation Program was notified on March 11, 2026 [1]. The *Caribbean Princess*, which departed Port Everglades on April 28, 2026, reported 160 cases of gastrointestinal illness during its 13-day Southern Caribbean voyage among 4,247 passengers and crew, corresponding to a 3.77% attack rate [2]. The *Ambition*, operated by Ambassador Cruise Line, departed Belfast on May 8, 2026, for a 14-night voyage to France and Spain. The vessel carried 1,187 passengers and 514 crew members (1,701 total). After arriving in Bordeaux, French authorities temporarily restricted disembarkation because of an outbreak of gastrointestinal illness. As of May 14, 2026, 60 passengers and four crew members were reported to be experiencing gastrointestinal illness, and laboratory testing confirmed norovirus [3].

**Table 1:** Characteristics of reported norovirus outbreaks on cruise ships from March to May, 2026

Vessel	Voyage dates	Total onboard	Reported cases	Attack rate	Key symptoms
Star Princess [1]	March 7–14, 2026	5,868	193	3.29%	Diarrhoea, vomiting
Caribbean Princess [2]	April 28–May 11, 2026	4,247	160	3.77%	Vomiting, diarrhoea
Ambition [3]	May 8–22, 2026	1,701	64	3.76%	Vomiting, diarrhoea

Norovirus is uniquely adapted for transmission aboard cruise ships. Its very low infectious dose, prolonged environmental persistence, resistance to commonly used disinfectants, and efficient aerosolisation during vomiting make it particularly suited to cruise ship environments [2,5]. Hundreds of passengers share ventilation systems, dining halls, recreational facilities, and semi-enclosed cabin corridors for prolonged periods. The CDC Vessel Sanitation Program continues to document recurrent gastrointestinal illness outbreaks aboard cruise ships, with norovirus identified as the causative agent in many reported events [6].

Regular handwashing, thorough cooking of shellfish, proper washing of fresh produce, and disinfection of contaminated surfaces are recommended preventive measures against norovirus [6]. Recent modelling studies further highlight the importance of early case identification and isolation during norovirus outbreaks aboard cruise ships. Analysis of seven Mediterranean cruise outbreaks involving 359 cases demonstrated that isolation of diagnosed individuals immediately upon symptoms reduced their transmissibility by approximately 94% under the study conditions. The same modelling framework improved forecasts compared with a baseline model, with improvement observed for 78% of data points at forecasting horizons longer than 72 hours [7]. These findings support rapid diagnosis and cabin isolation as important outbreak-control measures while also illustrating how mathematical modelling can complement routine syndromic surveillance.

There are still significant gaps in outbreak surveillance. Most cruise ships continue to rely primarily on symptomatic passengers reporting to onboard medical services, a strategy that frequently detects outbreaks only after substantial transmission has already occurred [8]. Routine sequencing of norovirus strains is rarely performed onboard, making it difficult to distinguish between single-source introduction, ongoing environmental contamination, foodborne transmission, and the emergence of novel recombinant variants. Genomic surveillance could additionally help track inter-voyage viral spread across ports and identify globally circulating strains associated with repeated maritime outbreaks [5].

This limitation has important public health implications. During outbreaks at sea, clinicians cannot readily determine whether cases result from a single introduction, ongoing environmental contamination, foodborne transmission, or the emergence of a new variant. Syndromic surveillance alone is insufficient to distinguish between these scenarios, although each requires different public health interventions [4,5,8].

Wastewater-based surveillance offers a practical solution. It requires no active passenger participation, can be integrated into existing ship infrastructure, and has already proven valuable as an early-warning tool during the COVID-19 pandemic. Wastewater surveillance could serve as a complementary, non-invasive early-warning approach on cruise ships and could be paired with genomic sequencing to characterize circulating norovirus strains. Passive wastewater sampling has been explored as a scalable infectious-disease surveillance strategy, including during the COVID-19 pandemic [9]. Such an approach could enable earlier detection while complementing, rather than replacing, clinical surveillance. Despite growing global attention to marine health security, these approaches remain largely absent from routine cruise ship operations.

Integrating syndromic surveillance with targeted wastewater-based molecular detection and genomic sequencing could provide a more comprehensive approach to maritime infectious-disease surveillance. The objective should not be to create an entirely new surveillance system onboard every vessel, but to complement existing medical reporting and sanitation systems with targeted molecular surveillance that is feasible, scalable, and linked to established public health response pathways. Such integration could facilitate earlier recognition of outbreaks, improve understanding of transmission, and strengthen preparedness for recurrent norovirus events associated with international cruise travel.
